# Recent technological advances in the management of chronic wounds: A literature review

**DOI:** 10.1002/hsr2.641

**Published:** 2022-05-19

**Authors:** Benson G. Ongarora

**Affiliations:** ^1^ Department of Chemistry Dedan Kimathi University of Technology Nyeri Kenya

**Keywords:** alternative medicine, biodegradable dressings, chronic wound, drug resistance.

## Abstract

**Background:**

Wound treatment comprises a substantial portion of the healthcare budgets in developed countries. Studies suggest that about 50% of patients admitted to hospitals have wounds, while 1%−2% of the general population in the developed world suffers from chronic wounds. Chronic wounds fail to repair themselves within the expected period of 30 days. Technologies have been developed to address challenges encountered during wound care with the aim of alleviating pain, promoting healing, or controlling wound infections.

**Objective:**

The objective of this study was to explore the technological improvements that have been made in this field over time.

**Methods:**

To gain insight into the future of wound management, a systematic review of literature on the subject was conducted in scientific databases (PubMed, Scopus, Web of Science, Medline, and Clinical Trials).

**Results and Discussion:**

Results indicate that wound dressings have evolved from the traditional cotton gauze to composite materials embedded with appropriate ingredients such as metal‐based nanoparticles. Studies on biodegradable dressing materials are also underway to explore their applicability in dressing large and irregular wounds. On the other hand, conventional drugs and traditional formulations for the management of pain, inflammation, infections, and accelerating healing have been developed. However, more research needs to be carried out to address the issue of microbial resistance to drugs. Drugs for managing other ailments also need to be designed in such a way that they can augment wound healing. In addition, it has been demonstrated that a coordinated integration of conventional and traditional medicine can produce laudable results in chronic wound management.

**Conclusion:**

Accordingly, collaborative efforts and ingenuity of all players in the field can accelerate technological advances in the wound care market to the benefit of the patients.

## Key messages

- Wounds affect about 50% of patients admitted to hospitals.

- Technologies have been developed including biodegradable dressing materials to address underlying challenges.

- Technological advancement, rising incidences of chronic wounds, growing government support, and a rising elderly population will drive wound market growth.

- A careful combination of recent research outputs can greatly change wound care technologies.

- This review highlights the recent research advances and opportunities in the wound care field.

- The future lies in biodegradable dressing materials, probably embedded with selected nanoparticles and which shall be combined in predetermined ratios.

## 1 BACKGROUND

Wounds are any disruptions or injuries caused to anatomical structures and functions, resulting from severe breakage in organs such as the skin.^1^ The damage can extend to other structures, including muscles, tendons, nerves, vessels, and bone. Wounds are generally classified as acute or chronic. Acute wounds are associated with surgical procedures, bites, burns, minor cuts and abrasions, severe traumatic lacerations, and gunshot injuries among other causes. Acute wounds normally heal within 1 month (4 weeks) through four spatially overlapping phases namely hemostasis, inflammation, proliferation, and remodeling.^2^, ^3^ Failure to proceed through these phases results in chronic wounds which are usually associated with high costs.^4^ In addition, based on the extent of the damage, such injuries are linked to increased morbidity and mortality.^5^

Chronic wounds are classified as vascular, diabetic, or pressure ulcers (PUs).^6^ All these ulcers are characterized by adamant infections, prolonged inflammation, presence of drug‐resistant microbial biofilms, and nonresponsive dermal or epidermal cells to reparative stimuli.^7^, ^8^, ^9^ The wounds mainly result from an influencing condition, which ultimately compromises dermal and epidermal integrity of tissues.^10^ Bacterial strains such as *Pseudomonas aeruginosa, Escherichia coli*, and *Staphylococcus aureus—*commonly associated with the human body*—*are the predominant causes of wound infections that result in delayed healing.^11^

The ulcers may also be a consequence of peripheral vascular disease, impaired venous drainage, or metabolic diseases such as diabetes mellitus.^12^, ^13^ Advancing age, poor nutrition, and immunosuppression associated with diseases including acquired immunodeficiency syndrome (AIDS), or treatment procedures, including chemotherapy or radiation therapy, can also lead to chronic ulcers.^14^, ^15^, ^16^, ^17^ However, pressure or decubitus ulcers result from continual compression of the skin. These ulcers occur on the hips, elbows, sacral region, and heels among other body parts.^18^, ^19^

Various technologies have been developed to address the challenges encountered during the management of such wounds. Topical therapies or dressings have been used in this pursuit. Sterile saline, cadexomer iodine, hypochlorous acid, honey, and superoxidized solution are some of the topically used agents.^20^, ^21^ The commonly used dressing materials range from standard cotton gauze to acrylics, honey alginates, hydrofibers, and hydrocolloids. Dressings that incorporate silver as an ingredient are mainly used to reduce microbial colonization,^22^ with short‐term benefits in wound‐healing, thus, prompting more studies on the role of metals in this process. Furthermore, promising recent experimental findings such as sprayable biodegradable materials^23^ have been reported, but they are yet to be explored in clinical trials.

On the contrary, some medical procedures such as chemotherapy deploy drugs that are specifically designed to inhibit cellular metabolism, rapid mitosis, and angiogenesis. Unfortunately, the inhibition affects many pathways such as DNA, RNA, or protein synthesis, which are critical in wound repair.^24^ In this review, the technological advances in wound dressings and management drugs have been evaluated. Experimental outputs from recent studies are also highlighted for possible trials at the clinical stage or for further laboratory studies.

## 2 METHODS

**Figure 1:**
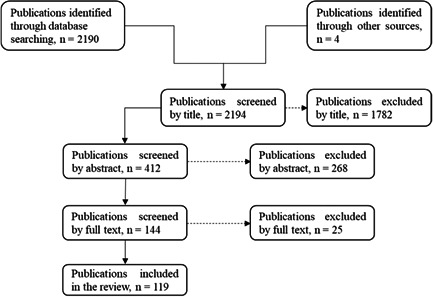
Flowchart showing the literature selection process for this article.

A literature review was conducted in peer‐reviewed databases, which include PubMed (https://pubmed.ncbi.nlm.nih.gov), Clinical Trials (https://www.medline.com), Scopus (https://scopus.com), Web of Science (https://www.webofscience.com/wos/woscc/basic-search), and Medline (https://www.medline.com), with a bias toward articles published after the year 2000. The following keywords were used: alternative medicine, biodegradable dressings, chronic wound, and drug resistance. A systematic review of chronic wound management technologies was carried out with the aim of identifying the existing gaps and the growth opportunities. The literature screening process for this study was carried out as shown in Figure 1, in which a large number of articles (*N* = 2194) were retrieved.

As selection criteria, all articles published in nonscientific journals and conferences were excluded. Duplicate articles and recalled articles were also excluded. In addition, articles published in other languages other than English were not considered in this study. Consequently, the number of articles decreased to 119. The outcomes obtained from recent research studies and the documented information as a result of recent literature reviews were then extracted, analyzed, and discussed in a manner that could help the readers (other researchers) to discern the potential of the wound care market. All the reviewed works were referenced in accordance with research ethical principles.

## 3 RESULTS AND ANALYSIS

This review yielded 119 publications that were relevant to chronic wound care technologies. A majority of the articles addressed the subject of interest as presented below, along with a discussion of the findings under the respective subtitles.

### 3.1 Economic and social burden of chronic wounds

To appreciate the magnitude of the challenges faced by the societies world over, an appraisal of the economic and social burden associated with wounds was done. An estimated prevalence rate of chronic wounds was found to range between 1% and 2% of the general population in developed countries,^25^ making them a significant burden to the patients, caregivers, and the medical system.^26^ In the United States, for example, about 8.2 million Medicare beneficiaries had at least one type of chronic wound or infection in the year 2014. A retrospective analysis of the data availed estimated that Medicare costs for all wounds ranged from $28.1 to $96.8 billion, including costs for infection management. Even though, the true cost of wound care remains unknown among the national population as well as the Medicare beneficiaries in the United States.^27^ Studies have projected that the advanced wound care market aiming at surgical wounds and chronic ulcers is likely to reach $15−$22 billion by 2024.^28^ This expansion is expected to be driven by technological advancement, rising incidences of chronic wounds, growing government support, and a rising elderly population.

Until the year 2000, the United States and most developed countries offered wound care procedures in the hospital setup. However, many nonhealing wounds were thereafter treated as outpatient cases. The United States, for example, had created approximately 1500 Centers for Medicare and Medicaid Services, which are hospital‐based outpatient payment systems. The primary goal of these centers was to provide care to patients who did not require hospitalization.^25^ Also, wound care services were, and still are, provided to patients at home through home health services and in skilled nursing facilities. This implies that the burden of care now shifts to the patients' relatives. Besides this, a review of the challenges faced in the treatment of chronic wounds demonstrated the inadequacy of evidence in terms of efficacy for a majority of the currently considered wound care products,^21^ implying that families are left to battle with medical challenges that would last for longer periods.

### 3.2 Management and treatment of wounds

Right from the onset, wound handling requires a thorough assessment of the patient and the wound itself to determine the appropriate measures which must be taken for favorable outcomes.^6^ For example, diabetic patients need deliberate control of their hyperglycemia, nutrition, and renal insufficiencies that are known to impair the wound healing process. These patients are vulnerable to diabetic foot ulcers (DFUs) that are difficult to manage. Other patients may be prone to venous leg ulcers or PUs, which are likely to cause other medical complications such as amputations. Therefore, holistic management of the patient is recommended to run simultaneously with the wound care therapies to optimize recovery. Underlying medical conditions could be managed through improved nutrition besides the prescribed medication. In addition, about 50% of the patients admitted to hospitals are malnourished,^29^ thus requiring proper assessment and subsequent recommendation of dietary supplements. Diet supplies the necessary ingredients for tissue repair.

Chronic wounds are normally expected to proceed to heal completely after 3 months if they attain a 50% reduction in terms of the area by the fourth week of occurrence.^30^, ^31^, ^32^ Any inconsistency with this prediction, especially where there are no underlying conditions, calls for an automatic reassessment of the wound and subsequently applying advanced therapies.^33^ Some of the advanced wound care technologies that have been developed include bioengineered allogeneic cellular therapies, stem cell therapies, xenograft cellular matrices, and growth factors.^21^ However, the selection of these advanced therapies depends on available evidence and it is recommended for use only when patients fail to respond to a normal healing curve.

Technologies for wound care that are espoused herein have been categorized into dressings and medications, with the aim of pointing out the inherent capabilities applicable to each case. However, in practice, these technologies are integrated during the management of chronic wounds.

### 3.3 Dressing materials

As mentioned earlier, the traditional dressing materials for wounds include the standard cotton gauze, acrylics, honey alginates, hydrofibers, and hydrocolloids. Nonetheless, dressing materials impregnated with nanocrystalline silver have been developed and used due to their improved wound healing abilities.^34^, ^35^ Similar results have been reported in a study that involved patients with burn wounds, where silver foam dressing relieved the patients of pain through infection control and rapid wound healing.^36^ A recent study involving silver‐releasing foam dressings versus silver‐containing cream in managing outpatients with DFUs also found that silver‐dressing foam is effective, especially during the early stages of wound management.^37^

A combination of silver with magnesium in Ag‐Mg‐thermoplastic polyurethane (Ag‐Mg‐TPU) composite material has correspondingly been shown to increase both the amount and rate of silver ion released from the composite, and with an increased antimicrobial effect.^38^ More recent advances in wound management technologies point to a brighter future for the healthcare sector and patients. A thiolated biodegradable bandage impregnated with ZnO nanoparticles has been found to improve tissue regeneration and it also hastens healing.^39^ Development of a biodegradable bilayer wound dressing material comprising polyurethane and ethanol extract of propolis (PU/EEP) on top of polycaprolactone gelatin (PCL/Gel) has equally added inspiration to future research on wound dressings.^40^ The material has been shown to possess desirable mechanical and antibacterial properties, it accelerates wound closure, and it is also biocompatible.

### 3.4 Medications

The findings in this category are further classified either as traditional or conventional interventions. One study reported an integrated approach to the management of chronic wounds. This case is presented independently under integrated interventions.

### 3.5 Traditional interventions in wound management

Many communities managed wounds traditionally using different approaches and based on the knowledge passed down from one generation to another.^41^ These developed interventions are referred to as traditional medicine. Traditional medicine refers to the health practices, approaches, knowledge, and beliefs incorporating plant, animal, and mineral‐based medicines, spiritual therapies, manual techniques, and exercises, applied singularly or in combination to treat, diagnose and prevent illnesses, or maintain well‐being.^42^ Patients resort to using these approaches mainly due to unaffordability or inaccessibility of alternative interventions,^43^ or because conventional interventions have not produced the expected outcomes.^44^, ^45^

Several reviews on the use of traditional wound management interventions have been published.^41^, ^46^, ^47^, ^48^, ^49^ The main aim of such reviews is to try to understand the contribution of traditional practices in addressing the problem at hand, and which has kept evolving with advances in technology. Some of the herbal interventions used against chronic wounds, including a Chinese herbal ointment, are effective and with less or no side effects.^50^, ^51^ Excellent results have been reported on wound care products, for instance, *Hibiscus sabdariffa*, Aloe vera, and honey.^48^, ^52^, ^53^ However, some of the traditional interventions may cause adverse effects on interacting with other products.^54^, ^55^

Chinese herbal medicine that has been accepted in the western countries as a topical ointment, WinVivo Healing Balm, was found to be useful in the management of difficult‐to‐heal lower extremity ulcers.^56^, ^57^ In China, it is used for treating second‐ and third‐degree burns.^58^ The ointment has also been used to treat acute and chronic wounds. It constitutes 11 main ingredients, including camellia oil and extracts of tree peony bark, *Coptis* root, lithospermum root, chicken gizzard, borneol powder, rhubarb root, corydalis rhizome, dragon's blood resin, fritillaria bulb, and bone ash. In two cases involving patients with hard‐to‐heal wounds, one patient had a 94% recovery after 2 months of WinVivo application while the second one had a 91% recovery after 3.5 months.

Some of the ingredients in WinVivo ointment exhibit properties that could be beneficial in wound healing. For instance, Peony bark has analgesic, antioxidant, anti‐inflammatory, and antipyretic properties.^59^ *Coptis* root (*Coptis chinensis*) contains berberine alkaloids, which are well known for their antimicrobial and anti‐inflammatory effects. In vitro and in vivo studies have shown that the plant possesses a broad spectrum of antibacterial, antifungal, and antiviral activity.^60^, ^61^ Antimicrobial agents exhibit accelerated wound healing when used systemically or topically, leading to the antimicrobial activity being accepted as a mechanism of wound healing.^62^, ^63^, ^64^ However, lack of antibacterial activity in an extract does not imply wound‐healing inability.^52^

### 3.6 Conventional medicine in wound management

Some of the commonly used medications for the treatment of wounded patients (glucocorticoid steroids, nonsteroidal anti‐inflammatory drugs [NSAIDs], and chemotherapeutic drugs) are known to affect wound healing.^65^ Low‐dosage of topical corticosteroid treatments is known to produce positive effects in chronic wounds. It accelerates healing, and reduces pain, besides suppressing hypergranulation tissue formation in 79% of the patients.^66^ However, systemic glucocorticoids (anti‐inflammatory agents) exhibit global anti‐inflammatory effects and are also known to suppress normal cellular wound responses, such as fibroblast proliferation and collagen synthesis. On the other hand, systemic steroids are known to cause reduced wound contraction.^24^ Consequently, caution should be exercised in case of prolonged use of these drugs to avoid the potential danger of infecting the injured body part.

NSAIDs such as ibuprofen, indomethacin, and aspirin are used for the management of inflammation and pain. Low‐dosage aspirin has an antiplatelet function and it is, therefore, commonly used to prevent asymptomatic infections from progressing to clinical diseases.^67^ Clinical recommendations to discontinue NSAIDs other than aspirin for a perioperative period of between four and five times the half‐life of drugs are usually made to avoid antiplatelet effects. However, cardiac patients are maintained on low‐dose aspirin due to the severe risk of cardiovascular events. The study also suggested that the Methotrexate drug was likely safe for use perioperatively.

Although the short‐term negative effect of NSAIDs on wound healing in humans is not clear,^68^ a retrospective study involving 75 patients, following laparoscopic colorectal resection for colorectal cancer, revealed that 33 of these patients who received diclofenac (nonsteroidal anti‐inflammatory drug) in the postoperative period had higher cases of anastomotic leakages compared to 42 who did not receive nonsteroidal drugs (7/33 vs. 1/42, *p* = 0.018), respectively.^69^ Based on these findings and additional animal studies, Klein recommended that NSAIDs should be abandoned after colorectal resection with primary anastomosis. In animal models, systemic use of ibuprofen has demonstrated a reduction in numbers of fibroblasts, delayed epithelialization, and reduced wound contraction, which is likely to push acute wounds to chronic status.^70^, ^71^ Impaired angiogenesis resulting from NSAIDs has also been reported in animal models.^72^

Chemotherapeutic drugs are usually designed to inhibit cellular metabolism, rapid mitosis, and angiogenesis. Unfortunately, the inhibition affects many pathways, such as DNA, RNA, or protein synthesis, which are critical to wound repair.^24^ The drugs also delay cell migration to the wound, matrix formation, and collagen production, in addition to impairing the proliferation of fibroblasts.^73^ Similarly, chemotherapy induces neutropenia, anemia, and thrombocytopenia, leading to other side effects, such as reduced immunity, excessive bleeding, or exposure to infection.^17^, ^65^ Bevacizumab, for example, is an antibody fragment (angiogenesis inhibitor) that is designed to limit the blood supply to the tumor, thus reducing the tumor's growth ability. Such drugs complicate the wound healing process,^74^ and therefore should be taken at least 28 days after surgery.^75^

To date, no chemotherapy drug has been developed with features that promote wound healing. Cyclophosphamide (Cytoxan) and cisplatin (Platinol) are known to block the cell cycle by alkylating DNA nucleotides, therefore, complicating the healing process.^73^ Although cyclophosphamide is not known to cause delayed wound healing in humans, it has been shown to cause a decrease in wound tensile strength. On the other hand, cisplatin slows down the proliferative phase of a wound in animals. Doxorubicin (Adriamycin) is a chemotherapy drug designed to inhibit mitosis of keratinocytes and decrease collagen synthesis and has been shown to cause macrophage dysfunction in rats.^76^

### 3.7 Integration of ayurvedic and conventional interventions

A successful case study involving integrated wound management has been reported in literature.^77^ In the research, the patient was treated with an integrative approach that involved ciprofloxacin (conventional drug) and *Triphalā* (ayurvedic medicine). Ciprofloxacin, 500 mg, was given twice daily for 10 days, accompanied by compression wrapping of the wounded area and diuretics as conventional practice. An ayurvedic procedure involving the washing of the affected part with *Triphalā* (a formulation composed of *Emblica officinalis*, *Terminalia chebula*, and *Terminalia bellirica*) to reduce pain and infection was performed. The decoction was used daily together with the application of wound dressing with turmeric powder, neem bark powder, and Medi honey. Gauze and a two‐layered compression wrap were also applied. Ayurvedic protocols continued daily for a period of 6 weeks. Marma therapeutic strokes to the legs were performed by a practitioner once per week for 6 weeks to improve blood circulation and increased the reabsorption of pooled lymph fluid. In addition, nutrition instructions were given to the patient to improve metabolic and digestive transformation for the entire period. The results of the integrated therapy were impressive and without any adverse effects.

### 3.8 Debridement

Another important aspect in the healing of wounds is debridement, which is a part of active wound care. It involves the removal of necrotic tissue that may result in a serious health condition.^78^ Various methods of debridement are available, including surgical, autolytic, enzymatic, and biological debridement.^79^ Autolytic debridement uses the body's own enzymes and moisture beneath the dressing and has been demonstrated to be an invaluable form of debridement.^80^ However, enzymatic removal of damaged cells or tissue has been utilized widely in wound care, and it mainly uses proteases. The therapeutic activity of animal secretions, such as fish epithelial mucus,^81^ maggot secretory products,^82^ and snake venom^83^ have been explored.

Maggot therapy is slowly becoming acceptable among patients as a debridement procedure.^84^ The medicinal larvae secrete digestive enzymes that selectively dissolve necrotic tissue, disinfect the wound, and stimulate healing.^85^ A survey on maggot and conventional therapies was conducted and the former proved effective.^86^ Maggot‐treated wounds saw a 50% reduction in necrotic surface area (half‐debrided) in 9 days, whereas conventionally treated wounds did not reach that stage until the 29th day. Within 4 weeks, larvae‐treated wounds were reported to be completely debrided, whereas those treated conventionally for an average of 5 weeks were still covered with over 33% of necrotic tissue.

### 3.9 Role of metals in wound healing

Metals are known for their unique ability to interact with important functional groups of biological molecules.^87^ Due to this, metals of interest have been used for many years as antimicrobial agents and have exhibited good efficacy against planktonic cells and biofilms.^88^, ^89^ Studies to understand the direct or indirect role of metals, such as copper, iron, zinc, and manganese in humans and other animals have been carried out.^90^, ^91^, ^92^ These metals are either involved in enzymatic or cellular activities including keratinocytes differentiation and fibroblasts expression. The topical application of antioxidant iron chelators, kojic acid, and deferiprone, has been found to accelerate the healing process.^93^ Of the two ointments, deferiprone performed better than kojic acid due to its higher antioxidant and iron chelation abilities.

Oral zinc supplementation has been recommended for patients with complications such as venous ulcers.^94^ These patients are susceptible to zinc deficiency. A link between zinc deficiency and delayed wound healing has been documented, although the optimal methods and true benefits of zinc supplementation are not clear.^95^ Topical supplementation of copper and zinc has also been shown to accelerate healing and strengthening of the cicatricial tissue.^90^, ^96^

Metallic nanoparticle materials consisting of silver, gold, and selenium have also been evaluated for wound healing and infection control.^97^, ^98^, ^99^ The rate of releasing the nanoparticles, the metal present, and the size of the particle play an important role in their toxicity against microbes.^38^, ^100^ Higher toxicities exhibited by smaller nanoparticles have been associated with their ability to cross the cell membrane in addition to their large surface area to volume ratio. Technologies have been developed to allow for sustained release of optimal concentrations of silver in place of silver nitrate. Acticoat, a nanocrystalline silver dressing material, is capable of releasing silver for up to 7 days.^101^

On the other hand, some studies have been carried out to analyze metal concentrations or to profile important phytochemicals found in medicinal herbs.^102^, ^103^ Results from the most promising plants have shown that these plant materials contain metals that could as well be acting as ingredients in wound healing. Research involving *Satureja khuzistanica* extracts has revealed that the plant's phytochemicals can accelerate wound healing in rats.^104^ Since some members from this genus have been used internally,^105^ then the profiling of other members of the genus may inform the formulation of drugs for the management of ailments such as stomach ulcers. A recent review on biodegradable metals for oral and maxillofacial applications has already laid foundations for future clinical investigations involving oral use.^106^

### 3.10 Drug resistance and wound management

It has been reported that microbial resistance to drugs increases with technological advances in the management of wounds and this could negate any advances made in wound management.^107^ This resistance could be a result of wrong drug prescription, wrong dosage, or other forms of abuse by professionals or patients, especially in developing countries.^108^ Noncompliance to recommended dosage has been reported among patients, particularly after favorable response to therapy. Others stop taking medication on signs and symptoms subsiding.^109^ Self‐medication, including the use of traditional formulations, is also a common practice in developing countries leading to drug resistance.^110^ Cases in which human drugs are used in agronomy, either to treat diseases or promote animal growth, have been reported.^111^ When such drugs are used on food‐animals, for example, drug‐resistant bacteria can be the result. A case in point of multidrug resistant bacteria presence in livestock and processed food has been reported in literature.^112^ All these examples indicate that drug resistance is a major problem across the globe. Therefore, proper regulation of the distribution and use of drugs is required to adequately address this challenge.

## 4 DISCUSSION

It is clear that tremendous progress has been made in the search for solutions to the complex situations facing people living with chronic wounds. Drugs for relieving pain, inflammation, and control of infections have been formulated. Dressings impregnated with nanoparticles in form of metals or metal oxides have been designed and availed for the management of wounds. Debridement technologies are also improving with the enzymatic approach being the target of the future. An integrated approach has also been employed in wound management. However, gaps still exist, mainly in facilitating the integrated approach in many countries.^113^ This uncoordinated approach can slow down the unveiling of new technologies that are likely to stem from shared knowledge and experiences. As an illustration, phytochemicals from ayurvedic medicinal herbs could be profiled and the obtained data applied thereafter in the designing of phytochemicals‐impregnated wound dressing materials.

Metals have been studied and their role in wound healing is increasingly becoming clearer. The demonstration of the elevated metal concentrations around the wound area during the healing process is an important development that should be explored further.^114^ Additionally, a sprayable and biodegradable material impregnated with or without AgNO_3_ that possesses antimicrobial properties has been developed.^23^ This material exhibited promising results in wound healing and control of infections during animal studies. It also has the potential to reduce the rate of changing the dressing material by 25% and with greater exudate absorption. Technologies incorporating these innovations are yet to be deployed as wound care strategies at the clinical level.

A correlation between mineral content in medicinal herbs that are topically used in wound management and the metal accumulation around the wound area is yet to be established, if any. Such information can guide the development of optimal metal‐impregnated wound dressing materials. Further, this insightful information can be combined with the knowledge that has been generated on biodegradable dressing materials. A sprayable biodegradable material has been developed and this may be fine‐tuned for possible application on large and irregular wounds that are usually difficult to dress. Wound dressings should be both comfortable and conformable.^115^ Debridement technology is also improving. Collagenase enzymatic debridement procedure can be utilized in making smart materials. The material could be sprayable and biodegradable, embedded with desired metals or phytochemicals, and finally with collagenase enzyme to help in the debridement process. Multi‐layering approach could perhaps be investigated.

A multipronged approach to the management of diabetic wounds has been evaluated.^116^ The review article analyzed the combination of metal nanoparticles with selected biomaterials and found that these materials can halt the growth and multiplication of bacterial strains associated with diabetic wounds. The composite also exhibited enhanced wound healing during in vitro and in vivo studies. Nevertheless, metal nanoparticles are associated with toxicities, which are mainly related to the method employed during synthesis and the characteristics of the nanoparticles. Similarly, toxicity as a result of topical application of silver formulations following its absorption through the wound is a matter of concern.^117^ Silver cytotoxicity against keratinocytes and fibroblasts has been found to occur at concentrations higher than 33 ppm.^118^

Therefore, the design of such materials will require combined efforts and a holistic understanding of the delicate balance that exists in the development of drugs and dressing materials. The patients must also understand their responsibility in the use of medication to avoid challenges associated with noncompliance in terms of under‐ or over‐dosage, misuse, and wrong use. Health practitioners play a critical role in the prescription and administration of drugs. Availability of drugs over the counter in itself is a risk to drug use. Standardization of traditional formulations or extracts and control of their prescription are equally important in the fight to curb drug resistance. Governments across the world need to put in place legislation to govern the proper use of drugs. Funding should also be availed to facilitate multidisciplinary research teams to address the challenges facing patients with wounds.

Finally, chemists and pharmaceutical companies need to fully appreciate the factors responsible for progressive wound healing such that they are able to design and synthesize drugs with features that will complement this process. As demonstrated in this review, drugs such as those used for chemotherapy have a counterproductive effect on the regeneration of the damaged tissue. Interruption of a patient's medication schedule with a view to undergoing surgery or due to an injury will definitely have its ramifications on their health. Among the listed drugs for suspension could be painkillers. Such a suspension would have a devastating effect on the patient including stress. Stress is a factor responsible for delayed wound healing and, therefore, counterproductive.^119^

## 5 CONCLUSION

Based on the research design, it is evident that chronic wounds affect a sizeable population in the developed world, and data from developing countries is scanty. It is also clear that significant progress has been made in terms of technological interventions toward the management of these wounds. Dressing materials, such as standard cotton gauze and nanoparticle‐embedded materials, are available to patients suffering from chronic wounds and wound care experts. In addition, traditional medicinal formulations and conventional drugs, such as ibuprofen, indomethacin, and aspirin have been developed for the therapeutic management of chronic wounds. Debridement procedures are equally advancing. Evidence also indicates that an integrated approach to the management of chronic wounds is a novel idea. These notwithstanding, challenges still abound, including drug resistance, high cost of drugs, inaccessibility of the existing technologies, technological shortcomings, and limited collaboration among stakeholders. Increased collaboration and research activities in traditional and conventional frontiers aimed at improving the existing technologies, and interventions are seemingly inevitable. Such technological advances, rising incidences of chronic wounds, growing government support, and a rising elderly population will drive wound market growth. In addition, further research is recommended on this topic since the current research design excluded articles written in languages other than English.

## AUTHOR CONTRIBUTION

Benson Ongarora independently planned and carried out this literature review. Five anonymous reviewers critiqued the draft article and gave insightful suggestions, which have been considered in the final version of the manuscript.

## CONFLICT OF INTEREST

The author declares no conflict of interest.

## TRANSPARENCY STATEMENT

This manuscript presents an accurate, honest and transparent analysis of all the articles included in the study. All the inclusion and exclusion criteria are provided and justified.
